# Severe inhibition of lipooligosaccharide synthesis induces TLR2-dependent elimination of *Mycobacterium marinum* from THP1-derived macrophages

**DOI:** 10.1186/s12934-017-0829-z

**Published:** 2017-11-28

**Authors:** Izabela Szulc-Kielbik, Jakub Pawelczyk, Michal Kielbik, Laurent Kremer, Jaroslaw Dziadek, Magdalena Klink

**Affiliations:** 1 Institute of Medical Biology, Polish Academy of Sciences, 106 Lodowa Str., 93-232 Lodz, Poland; 2 IRIM (ex-CPBS)-UMR 9004, Research Institute of Infectiology of Montpellier, Université de Montpellier, CNRS, 34293 Montpellier, France; 3 INSERM, IRIM, 34293 Montpellier, France

**Keywords:** *Mycobacterium marinum*, LOS, THP-1-derived macrophages, TLR2, Infection

## Abstract

**Background:**

Although mycobacterial glycolipids are among the first-line molecules involved in host–pathogen interactions, their contribution in virulence remains incomplete. *Mycobacterium marinum* is a waterborne pathogen of fish and other ectotherms, closely related to *Mycobacterium tuberculosis*. Since it causes tuberculosis-like systemic infection it is widely used as a model organism for studying the pathogenesis of tuberculosis. It is also an occasional opportunistic human pathogen. The *M. marinum* surface-exposed lipooligosaccharides (LOS) are immunogenic molecules that participate in the early interactions with macrophages and modulate the host immune system. Four major LOS species, designated LOS-I to LOS-IV, have been identified and characterized in *M. marinum*. Herein, we investigated the interactions between a panel of defined *M. marinum* LOS mutants that exhibited various degrees of truncation in the LOS structure, and human-derived THP-1 macrophages to address the potential of LOSs to act as pro- or avirulence factors.

**Results:**

A moderately truncated LOS structure did not interfere with *M. marinum* invasion. However, a deeper shortening of the LOS structure was associated with increased entry of *M. marinum* into host cells and increased elimination of the bacilli by the macrophages. These effects were dependent on Toll-like receptor 2.

**Conclusion:**

We provide the first evidence that LOSs inhibit the interaction between mycobacterial cell wall ligands and appropriate macrophage pattern recognition receptors, affecting uptake and elimination of the bacteria by host phagocytes.

## Background

*Mycobacterium marinum* is a waterborne pathogen that is phylogenetically related to *M. tuberculosis* and causes tuberculosis-like systemic infection in fish and in other ectotherms [1–3]. It can also induce granulomatous infection in humans called “fish tank disease” [2] and, as other nontuberculous mycobacteria, is responsible for opportunistic infections in immune-deficient patients [4]. Similar to *M. tuberculosis, M. marinum* is able to replicate and survive within infected host cells. As both species share a conserved skeleton of host–pathogen interactions, *M. marinum* is also widely used as a surrogate to decipher many aspects of the immunopathogenesis of tuberculosis [5]. Therefore it is vital to gain insight into the structure and biological significance of cell-envelope associated molecules that may be important for *M. marinum* immunopathology.

The uptake of mycobacteria by professional phagocytes, such as macrophages, is dependent upon the early recognition of the pathogen-associated molecular patterns (PAMPs) by specific pathogen recognition receptors (PRRs) that are crucial in initiating and driving the host immune response. Among the PRRs, Toll-like receptor 2 (TLR2) and complement receptor 3 (CR3) have been demonstrated to play key roles in macrophage-*Mycobacterium* interactions [6]. Among the PAMPs, phosphatidyl-*myo*-inositol mannosides (PIM), lipomannan (LM), lipoarabinomannan (LAM), lipoproteins or proteins, such as the Ag85 complex, have been documented to act as TLR2 and/or CR3 ligands in the early events that precede phagocytosis by macrophages [7–9].

A major hallmark of mycobacteria is the very thick and highly impermeable cell envelope, which plays a critical role in innate resistance to many antimicrobial agents and in directing host–pathogen interactions [10]. Although the structure, biosynthesis and physiological roles of the cell wall-associated mycolyl-arabinogalactan-peptidoglycan (mAGP) core complex have been well described [11–13], our knowledge regarding the (glyco)lipids interspersed within the mAGP remains incomplete in many aspects. These extractable lipids largely contribute to the modulation of the host immune system and in conditioning infection outcomes [14, 15]. They comprise the highly polar, surface-exposed lipooligosaccharides (LOSs) that have been reported in more than 10 mycobacterial species, such as *Mycobacterium marinum*, *Mycobacterium kansasii*, *Mycobacterium gastri, Mycobacterium szulgai* and the *Mycobacterium canettii* variant of *Mycobacterium tuberculosis* [16–22]. Among the different species, these glycolipids exhibit considerable structural variations in the glycan core as well as in the lipid moiety.

LOSs are of great interest, as it has been inferred that they are structurally and functionally similar to other trehalose-containing glycolipids, such as diacyltrehalose (DAT) and polyacyltrehalose (PAT) in *M. tuberculosis* [23]. In *M. marinum,* four major LOS species, designated LOS-I to LOS-IV, have been identified and characterized [24]. The structure of LOS-I comprises a glycan core consisting of trehalose, glucose and one methylated rhamnose (3-*O*-Me-Rha*p*-(1-3)-Glc*p*-(1-3)-Glc*p*-(1-4)-Glc*p*-(1-1)-Glc*p*). This oligosaccharide moiety is subsequently glycosylated by additional monosaccharides, giving rise to more polar LOS species. In addition to the glycan core, LOS-II, LOS-III and LOS-IV are substituted by α-d-Xyl*p*. LOS-II also possesses a terminal α-caryophyllose residue (α-3,6-dideoxy-4-*C*-[d-*altro*-1,3,4,5-tetra-hydroxyhexyl]-d-*xylo*-hexopyranose), whereas LOS-III and LOS-IV contain two α-caryophyllose units [25]. The second caryophyllose of LOS-IV is decorated by an atypical N-acetylated dideoxy galactose [26]. Similar to other trehalose-based glycolipids, all LOS species are acylated by polymethyled fatty acids [27]. Depending on growth medium, various LOS subspecies exist in a different amount and proportion [24], indicating that *M. marinum* has the ability to modulate its LOS content.

LOSs are known as highly antigenic glycoconjugates exposed to the cell surface [15]. In *M. marinum*, they have clearly been associated with colony morphology, sliding motility and biofilm formation [28]. Although early studies suggested their involvement in macrophage uptake [25, 28], their contribution to the virulence and pathogenesis of *M. marinum* remains obscure. Initial work on *M. kansasii* indicated that rough variants devoid of LOSs induce chronic infections, whereas smooth variants producing LOSs are rapidly eliminated [29, 30], leading to the hypothesis that LOSs may be considered as avirulence determinants. The work of Alibaud et al. [31], based on a large panel of selected/generated LOS mutants led to the observation that phagocytosis of *M. marinum* was conditioned by the LOS pattern and pointed to an inverse correlation between LOS production and uptake by J774 murine macrophages or amoeba, which contrasted with another study in which LOS-deficient mutants were associated with impaired cell entry [28].

**Fig. 1:**
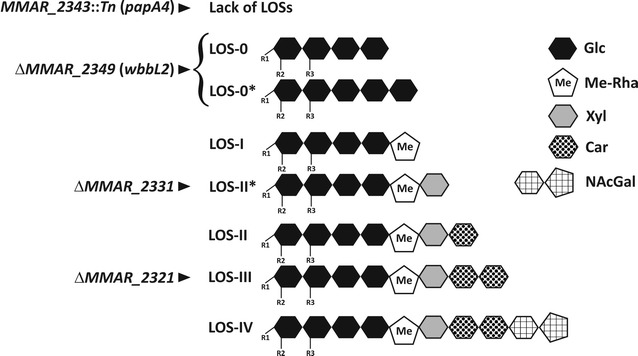
Schematic representation of the different LOS subspecies and intermediates synthesized by the *M. marinum* mutants. The genes inactivated in the different mutants are indicated. *Glc* glucose, *Me-Rha* methylated rhamnose, *Xyl* xylopyranose, *Car* caryophyllose, *NAcGal N*-acylated dideoxygalactose, *R1, R2, R3* the acyl chains

In this study, among a vast panoply of previously characterized *M. marinum* mutants [27, 31], producing different LOS variants due to mutations in the genes involved in the early or late stages of the LOS biosynthetic pathway, four were selected, each representing a particular step of LOS pathway inhibition (Fig. 1). Briefly, the Δ*MMAR_2321* mutant that lacked the probable *N*-acyltransferase involved in the linkage of the pyrrolidone cycle on the α-4-amino-4,6-dideoxy-Gal*p* residue of LOS-IV produced all LOS subspecies, except the most polar LOS-IV [31]. Disruption of *MMAR_2331* leads to a mutant that is unable to produce LOS-II, LOS-III and LOS-IV; however, it accumulates LOS-II*—a LOS-II intermediate lacking the terminal caryophyllose, thus connecting *MMAR_2331* to either the synthesis of caryophyllose or to its transfer on LOS-II* to produce LOS-II [31]. The Δ*MMAR_2349* (*wbbL2*) mutant is affected at a very early step in the LOS pathway, leading to the accumulation of LOS-0—a LOS-I precursor devoid of the terminal methyl-Rha residue, which confirms the involvement of WbbL2 in rhamnose metabolism. Additionally, the accumulation of LOS-0 also leads to the synthesis of the so-called LOS-0*, most likely through the addition of another glucose residue onto the tetraglucosyl backbone of LOS-0 [31]. Disruption of *MMAR_2343* (*papA4*) resulted in complete LOS breakdown, indicating that PapA4 fulfills the requirements for LOS acylation and assembly [27]. Described mutants were used to revisit the potential correlation between the LOS structure and mycobacterial macrophage infection.

## Results

### Phagocytosis of *M. marinum* LOS mutants by THP-1 macrophages

**Fig. 2:**
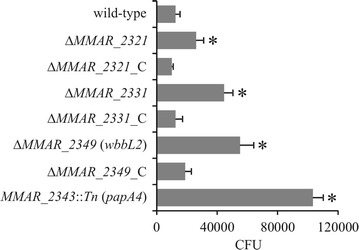
Phagocytosis of the *M. marinum* mutants by macrophages. Macrophages were incubated with *M. marinum* strains for 2 h at an MOI of 10. Non-ingested bacteria were extensively washed and killed by gentamycin. Macrophages were lysed with Triton X-100, and then cell lysates were plated onto Middlebrook 7H10 agar supplemented with 10% OADC for CFU determination. The number of ingested bacteria are presented as the mean ± SEM from 6 to 7 separate experiments. Figure describes the difference in phagocytosis between mutants, their complemented counterparts and wild-type *M. marinum*. Statistical significance: *p ≤ 0.03 (Mann–Whitney *U* test)

Previous studies suggested that the presence of LOS facilitates the uptake of *M. marinum* by macrophages [28], whereas other studies suggested that the *M. marinum* strains producing shorter LOS versions, were more efficiently phagocytosed by J774 murine macrophages or by *Acanthamoeba* [31]. To examine the role of LOS in the uptake of *M. marinum* by THP-1 macrophages, the number of bacteria ingested by macrophages was determined by CFU plating after a 2-h incubation period (Fig. 2). The ability of the wild-type *M. marinum* strain to enter macrophages is very low (only 1 ± 0.2% of phagocytosis). However, in contrast to the wild-type strain, the mutants exhibiting truncated LOS variants were more efficiently phagocytosed by THP-1 macrophages, which is consistent with a previous study performed in J774 cells [31]. Accordingly, the phagocytosis percentage increased progressively and correlated to the decrease in the LOS structure size: 3% ± 0.4, 4% ± 0.5 and 7% ± 0.8 in the Δ*MMAR_2321*, Δ*MMAR_2331* and Δ*MMAR_2349* (*wbbL2*) strains, respectively. Remarkably, this phenomenon was further enhanced in the LOS-deficient *MMAR_2343*::*Tn* (*papA4*) mutant (12% ± 1.0). All complemented control strains were phagocytosed similarly to the wild-type strain (Fig. 2). Overall, these results show that the presence of LOS imparts the bacterial phagocytosis efficiency by macrophages.

### Minimal LOS structure requirements for intramacrophage survival

**Fig. 3:**
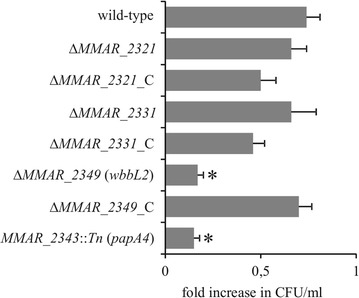
Intracellular survival of the LOS mutants in macrophages. Macrophages were incubated with *M. marinum* strains for 2 h at an MOI of 10. Non-ingested bacteria were extensively washed and killed by gentamycin. Macrophages were either lysed with Triton X-100 or maintained in culture for 2 additional days prior to lysis. Cell lysates were plated onto Middlebrook 7H10 agar supplemented with 10% OADC for CFU determination. The data are presented as fold increase in CFU/ml (number of bacteria 2 days post-infection divided by the number of bacteria after 2-h of phagocytosis), expressed as the mean ± SEM from 6 to 7 separate experiments. Figure describes the difference in intracellular survival between mutants, their complemented counterparts and wild-type *M. marinum*. Statistical significance: *p ≤ 0.05 (Mann–Whitney *U* test)

We next addressed whether the mutants were affected in their intramacrophage survival rate. The CFU counts after 48 h of growth in THP-1 macrophages resulted in a sharp decrease in the survival of the ∆*MMAR_2349* (*wbbL2*) and *MMAR_2343*::*Tn* (*papA4*) mutants compared with the wild-type strain (Fig. 3). However, the strains with less severe changes in their LOS profiles (Δ*MMAR_2321*, lacking LOS-IV or Δ*MMAR_2331*, lacking LOS-II to LOS-IV) grew similarly to the wild-type strain inside of the macrophages. These results suggest that near-complete LOS compounds are sufficient for optimal survival of *M. marinum* in their macrophage host.

### Impact of *M. marinum* on macrophage TLR2 and CR3 surface receptor expression

**Fig. 4:**
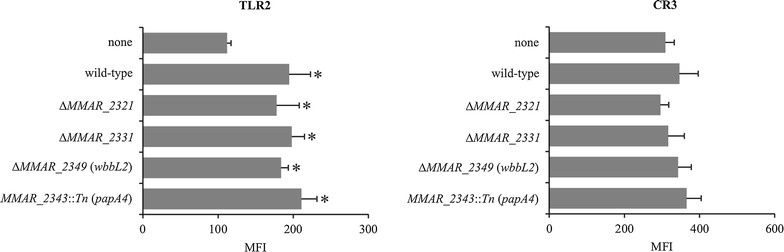
Impact of the *M. marinum* strains on TLR2 and CR3 expression. Macrophages were incubated with the various *M. marinum* strains for 2 h at an MOI of 10, after which the cells were stained with fluorochrome-conjugated antibodies for 30 min at 4 °C and analyzed by flow cytometry. The data are presented as the mean median fluorescence intensity (MFI) ± SEM from five separate experiments. For statistical significance *p ≤ 0.05 (Mann–Whitney *U* test) the following datasets were compared: uninfected macrophages versus macrophages infected with mutants or wild-type strain

TLR2 and CR3 are important cell surface recognition receptors for mycobacteria [6]; therefore, we next assessed the contribution of CR3 and TLR2 in the phagocytosis of the *M. marinum* strains. Macrophages were first infected with *M. marinum* and then stained with fluorochrome conjugated anti-TLR2 or anti-CR3 mAbs. Surface marker expression was then assessed by flow cytometry, and non-infected cells were included as controls. The median fluorescence intensity (MFI) of the non-infected macrophages amounted to 309 ± 24 and 112 ± 5 for CR3 and TLR2, respectively (Fig. 4). Infection of the cells for 2 h with either the wild-type or LOS mutant strains was accompanied by a significant increase in TLR2 expression—MFI = 195 ± 28 (wild-type), 178 ± 30 (Δ*MMAR_2321*), 198 ± 17 (Δ*MMAR_2331*), 184 ± 9 (*∆MMAR_2349*), 211 ± 21 (*MMAR_2343*::*Tn*). However, the CR3 expression level remained unaffected by the presence of *M. marinum* (Fig. 4). Together these results suggest that *M. marinum* induces the specific expression of TLR2, but not of CR3, in a LOS-independent manner.

### Involvement of TLR2 and CR3 in *M. marinum* phagocytosis

**Fig. 5:**
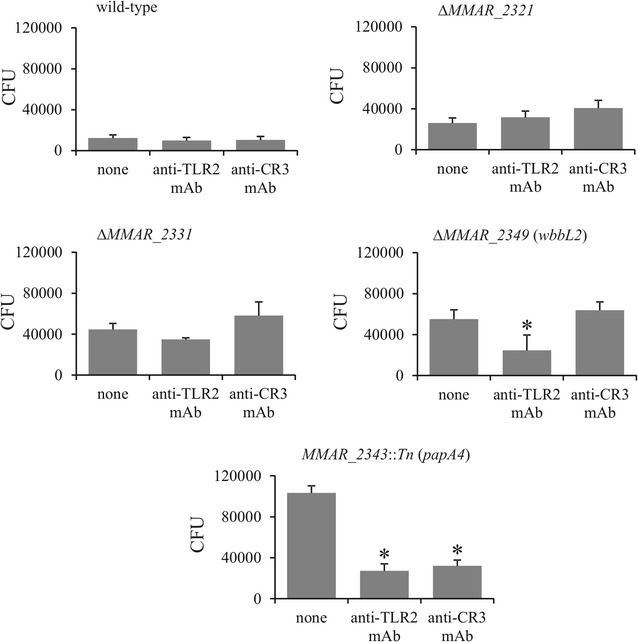
Involvement of TLR2 and CR3 in phagocytosis of the *M. marinum* strains by macrophages. Comparison of the *M. marinum* strains phagocytosis rates after blocking of macrophage TLR2 or CR3. Macrophages were pretreated with blocking antibodies (anti-TLR2 or anti-CR3) for 1 h and incubated with the *M. marinum* strains for 2 h at an MOI of 10. Non-ingested bacteria were extensively washed and killed by gentamycin. Macrophages were then lysed with Triton X-100 and the cell lysates were plated onto Middlebrook 7H10 agar supplemented with 10% OADC for CFU determination. The data are presented as the mean number of ingested bacteria ± SEM from six separate experiments. For statistical significance: *p ≤ 0.03, (Mann–Whitney *U* test) the following datasets were compared: mutant/wild-type versus mutant/wild-type + blocking antibodies

The abovementioned results prompted us to assess the contribution of CR3 and TLR2 in the phagocytosis of the *M. marinum* strains. Macrophages were first pre-treated with blocking anti-TLR2 or anti-CR3 mAbs prior to infection. CFUs corresponding to the ingested bacilli were then determined by plating the cellular lysates. The results clearly demonstrated that blocking of either CR3 or TLR2 strongly reduced the entry of the *MMAR_2343*::*Tn* (*papA4*) strain into the cells (Fig. 5). The ∆*MMAR_2349* (*wbbL2*) mutant ingestion was also dependent on TLR2 expression but not on CR3 expression. In contrast to the *MMAR_2343*::*Tn* and ∆*MMAR_2349* strains, blocking of either TLR2 or CR3 had no significant effect on the uptake of the Δ*MMAR_2321* and Δ*MMAR_2331* mutant or wild-type strains (Fig. 5). In control experiments we tested the phagocytosis of *M. marinum* strains by macrophages pre-treated with appropriate isotype control and we did not notice any influence on bacteria entry. The mean numbers of CFU/ml after a 2-h phagocytosis of wild-type and Δ*MMAR_2321,* Δ*MMAR_2331,* Δ*MMAR_2349* or *MMAR_2343*::*Tn* mutants were 18,790, 30,625, 43,125, 49,386, 119,913 in the presence of isotype control to anti-TLR2 mAbs and 12,356, 33,625, 42,863, 46,410, 93,040 in the presence of isotype control to anti-CR3 mAbs, respectively. Moreover we tested the influence of blocking antibodies on activity of non-infected macrophages. We found that PMA-induced ROS generation by macrophages in the presence or absence of anti-TLR2 or anti-CR3 mAbs was similar (RLU total = 1091; 1008; 915, respectively). Secondly, the nitric oxide production (measured as nitrite) was also similar (control macrophages = 0.4 µM; macrophages + anti-TLR2 mAbs = 0.39 µM; or + anti-CR3 mAbs = 0.33 µM). These results suggest that complete, but not near-complete, lack of LOS structures facilitates interaction of TLR2 with its cell wall agonists, which increases the entry of *M. marinum* into host cells.

### Participation of TLR2 and CR3 in the intramacrophage survival of *M. marinum*

**Fig. 6:**
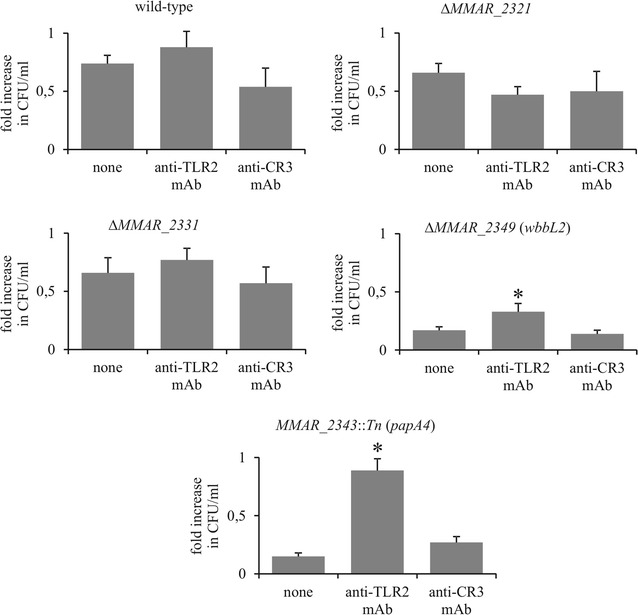
Involvement of TLR2 and CR3 in intracellular *M. marinum* survival. Macrophages were pretreated with blocking antibodies (anti-TLR2 or anti-CR3) for 1 h and incubated with *M. marinum* mutants for 2 h at an MOI of 10. Non-ingested bacteria were extensively washed and killed by gentamycin. Macrophages were lysed with Triton X-100 or maintained in culture for another 2 days and then lysed, plated on Middlebrook 7H10 agar supplemented with 10% OADC and cultured for CFU determination. The data are presented as fold increase in CFU/ml (number of bacteria 2 days post-infection divided by the number of bacteria after 2-h of phagocytosis), expressed as the mean ± SEM from six separate experiments. For statistical significance: *p ≤ 0.03, (Mann–Whitney *U* test) the following datasets were compared: mutant/wild-type versus mutant/wild-type + blocking antibodies

The potential contribution of TLR2 and CR3 expression on intramacrophage survival was next investigated following CFU determination of intracellular bacteria after pretreatment of the cells with the corresponding blocking mAbs. The growth comparisons of the wild-type strain and the LOS mutants indicate that blocking of TLR2, but not CR3, significantly increased the replication rate of both the ∆*MMAR_2349* (*wbbL2*) and the *MMAR_2343*::*Tn* (*papA4*) mutants (Fig. 6). TLR2 or CR3 blocking did not influence the intracellular growth of the wild-type strain or the Δ*MMAR_2321* and Δ*MMAR_2331* mutant strains. Overall, this indicates that the lack of LOS severely increases *M. marinum* elimination in a TLR2-dependent manner.

## Discussion

Although the LOS structures and their distribution have been well studied in various mycobacterial species, their roles in pathogenesis and virulence are still unclear [25, 28–30, 32]. By studying a large panel of defined *M. marinum* mutants with impaired LOS synthesis, in a previous paper Alibaud et al. [31] demonstrated in the J774 cell line that *M. marinum* phagocytosis is conditioned by the LOS pattern and pointed to a clear inverse correlation between LOS production and the efficient uptake into professional phagocytic cells. The mentioned results, together with other studies done with *M. marinum* [32] or *M. kansasii* [29, 30] seem to confirm the thesis of LOSs as factors masking the bacterial ligands that are recognized by host cells. However, neither the influence of LOS inhibition on *M. marinum* intramacrophage survival nor their “masking” potential in host–pathogen interactions has been clearly confirmed.

In this work, the interplay between *M. marinum* LOS mutants and human macrophage-derived THP-1 cells was explored to address the ability of these structures to mask other mycobacterial cell wall components which, in turn, may act as potential ligands for pathogen recognition receptors. The complete absence (*MMAR_2343*::*Tn*) or synthesis of only the early LOS subspecies (Δ*MMAR_2349*) resulted in highly enhanced uptake by macrophages. Analysis of the *MMAR_2343*::*Tn* and Δ*MMAR_2349* mutant intracellular survival revealed that both strains were effectively eliminated by the phagocytes. Conversely, the replication of two mutants with moderately impaired LOS structure (Δ*MMAR_2321*, lacking LOS-IV or Δ*MMAR_2331*, lacking LOS-II to LOS-IV) remained unchanged compared with the wild-type strain, despite the significantly increased phagocytosis rate.

To address how the various LOS profiles influence their interactions with phagocytes, we first analyzed the expression level of two major macrophage PRR in response to *M. marinum* infection. This resulted in a significant increase in TLR2 but not CR3 expression. Additionally, TLR2 blocking significantly reduced the uptake of the *MMAR_2343*::*Tn* (*papA4*) and Δ*MMAR_2349* (*wbbL2*) mutants and increased their survival within macrophages. Moreover, the blocking of CR3 receptor indicated that it could participate in another pathway of *MMAR_2343*::*Tn* uptake, despite the fact that its expression was not significantly increased upon infection. However, CR3 blocking did not affect the intracellular survival of *MMAR_2343*::*Tn*. This is consistent with the reported role of CR3 to act as a “silent receptor”, allowing pathogenic bacteria to enter into macrophages without inducing mycobacterial killing [33, 34]. Strikingly, the fates of Δ*MMAR_2321* and Δ*MMAR_2331* in the early infection events seem to be TLR2- and CR3-independent.

Our results add new insight into the role of LOS, which may mask other cell wall-associated PAMPs, thereby conditioning the outcome of the infection. Moderately impaired LOS structures failed to uncover antigenic determinants that may be recognized by their cognate macrophage receptors. The increased phagocytosis of Δ*MMAR_2321* and Δ*MMAR_2331* may therefore result from a modified cell surface composition/architecture. In example the lack of at least LOS-IV is sufficient to reduce the release of PE_PGRS proteins from the mycobacterial cell surface [32]. Moreover high number of other secretion mutants with disruption in LOS biosynthesis region ranging from LOS-IV to complete LOS deficiency show clear correlation between LOS content and PE_PGRS release [32]. Since the strongest secretion defect is observed in LOS deficient mutants, it seems like LOS may act as a detergent for capsular proteins. However, the absence of LOS-IV resulted in an altered surface attachment of PE_PGRS proteins, indicating that the terminal sugar moiety plays a role in this process [32]. PE_PGRS proteins are substrates for the ESX-5 secretion system and implicated in virulence [35–39]. Therefore, LOS may play a role in the modulation of mycobacterial virulence by regulation of surface protein attachment. Similar function was also attributed to diacyltrehalose (DAT) and polyacyltrehalose (PAT) of *M. tuberculosis* which are closely related to LOS. Phenotypic observations made on a mutant of *M. tuberculosis* deficient in the biosynthesis of DAT and PAT indicated a role for these lipids in the retention of the capsular material at the cell surface. Such modification of the mutant surface properties increased its binding and uptake by phagocytic and nonphagocytic cells, but did not influence its ability to replicate and persist in cultured macrophages and in mice [23].

In Δ*MMAR_2331,* not only LOS-IV but also LOS-III and LOS-II production is arrested [31]. Similar to Δ*MMAR_2332* [28] and Δ*MMAR_2333* [40], Δ*MMAR_2331* accumulates the LOS-II biosynthetic precursor (LOS-II*). The intracellular growth of these three Δ*MMAR_2331*-Δ*MMAR_2333* mutants was not attenuated; however, only in the case of Δ*MMAR_2331*, a concomitant increase in host cell entry was detected.

From our structural/functional relationship studies, it can be inferred that the minimal functional LOS structure is LOS-II. The results suggest that all mutants exhibiting an unchanged or improved ability to invade macrophages require at least the LOS-0 to LOS-II* structures. Deeper changes of the LOS structure, that goes beyond LOS-II, present in the Δ*MMAR_2349* (*wbbL2*) and *MMAR_2343*::*Tn* (*papA4*) mutants, exerts additional effects on the host–pathogen interaction and increases clearance of the pathogen from the host cell. Interestingly, the suggested functional LOS structure distinction corresponds to the LOS synthesis and transport. Sarkar et al. [40] proposed a model for LOS biosynthesis, wherein early intermediates up to acylated hexasaccharide comprised LOS-II* are synthesized intracellularly and then transported across the membrane. Further elongation of LOS-II* occurs on the extracytoplasmic site. We cannot exclude that by the precise regulation of extracytoplasmic glycosyltransferases, the mycobacterial cell is able to modify the final LOS structure in response to different environmental factors.

It appears that a complete or at least a near-complete LOS structure is required for survival in the host cell, and LOS-deficient strains are eliminated in a TLR2 dependent manner. Signaling through TLR2 is important for macrophage activation and induction of a protective immune response to mycobacterial infection [41]. The mycobacterial cell envelope contains many diverse TLR2 ligands, such as lipoproteins, lipoglycans and glycolipids [42]. Among the various mycobacterial glycolipids that interact with TLR2, only a limited number of TLR2 agonists have been reported in *M. marinum*. These include the apolar, surface-exposed dimycolyl-diarabino-glycerol (DMAG) [43]. Due to structural similarities between DMAG and other mycobacterial glycolipids that interact with TLR2 (e.g. trehalose dimycolate or glucose monomycolate), one can assume that the spectrum of molecules exposed/uncovered in the LOS mutants that promote TLR2-dependent activation is relatively broad. The phagocytosis of the *MMAR_2343*::*Tn* (*papA4*) mutant, in which LOS synthesis is completely blocked, appeared to be not only TLR2 but also CR3 dependent. The essential role of the abundance and composition of polysaccharides, especially PIMs, was demonstrated for the non-opsonic binding of mycobacterium to CR3 [44]. As the LOSs may be present across the whole polysaccharide-protein matrix of the mycobacterial cell wall [45], we suspect that their absence results in the reorganization and better exposure of polysaccharides that directly interact with CR3, thus promoting this phagocytosis pathway.

## Conclusions

Our data confirm the roles of different structural LOS variants in modulating *M. marinum* interaction with phagocytes. The absence of LOS results in an unmasking of cell wall surface epitopes that are recognized by macrophage receptors, which eventually lead to a greater elimination of the pathogen. We proposed that this effect might be mediated through the TLR2 pathway. The precise regulation of the lipooligosaccharide content in response to variable environmental conditions demands further study.

## Methods

### Reagents and antibodies

RPMI 1640 (Roswell Park Memorial Institute) medium, fetal bovine serum (FBS), Dulbecco’s phosphate buffered saline (D-PBS) and Hanks’ balanced salt solution (HBSS) were purchased from Life Technologies (Carlsbad, CA, USA). Middlebrook 7H10 agar, Middlebrook 7H9 broth and Middlebrook OADC enrichment were acquired from Becton–Dickinson (Franklin Lakes, NJ, USA). Phorbol-12-myristate-13-acetate (PMA), Triton X-100, gentamycin, β-mercaptoethanol, penicillin (10,000 U/ml)/streptomycin (10 mg/ml) solution (P/S), Tween 80 and 4% formaldehyde (FA) solution, horseradish peroxidase (HRP), luminol, sodium nitrite, were obtained from Sigma-Aldrich (St. Louis, MO, USA). Human type AB serum was purchased from PAN-Biotech GmbH (Aidenbach, Germany). Mouse IgG2a anti-human TLR2 (sodium/azide free) mouse IgG2a isotype control (azide free), phycoerythrin (PE)-conjugated mouse IgG2a anti-TLR2 and PE-conjugated mouse IgG2aκ isotype control antibodies were obtained from Novus Biologicals (Littleton, CO, USA). Mouse IgG1κ anti-human CR3 (CD11b/Mac-1; sodium/azide free) NA/LE mouse IgG1κ isotype control (sodium/azide free), PE-conjugated mouse IgG1κ anti-human CR3, PE-conjugated NA/LE mouse IgG1 isotype control antibodies were purchased from BD Biosciences (San Jose, CA, USA).

### Bacterial strains and culture conditions

The *Mycobacterium marinum* strain M was isolated from a human patient [3], and the *M. marinum* mutants—Δ*MMAR_2321*, Δ*MMAR_2331*, Δ*MMAR_2349* (*wbbL2*), *MMAR_2343*::*Tn* (*papA4*) and complemented strains - Δ*MMAR_2321*_C, Δ*MMAR_2331*_C, Δ*MMAR_2349*_C were reported previously [31] Cultures were grown/maintained at 30 °C in 7H9 liquid or 7H10 solid medium containing 10% oleic acid, albumin, dextrose and catalase (OADC) enrichment, supplemented with hygromycin (80 µg/ml) where necessary. The two-step homologous recombination protocol [46] was used to introduce unmarked deletions into the *MMAR_2321*, *MMAR_2331* and *MMAR_2349* (*wbbL2*) genes of *M. marinum* [31]. The *MMAR_2343*::*Tn* (*papA4*) strain was selected through a screen of a *M. marinum* transposon library to identify new cell wall-defective mutants [47]. The corresponding complemented strains, designated Δ*MMAR_2321*_C, Δ*MMAR_2331*_C and Δ*MMAR_2349*_C, were generated through the introduction a functional copy of the genes cloned either under the control of their own promoters (*MMAR_2331* and *MMAR_2349*) or the constitutive *hsp60* promoter (*MMAR_2321*) in the integrative pMV306 vector [31].

For use in infection tests, *M. marinum* wild-type, its mutants and complemented strains were grown in Middlebrook 7H9 broth enriched with 10% OADC and 0.05% Tween 80 for 7–10 days to reach an optical density of 1 (OD_600_). Then, the bacteria were divided into aliquots and stored at − 70 °C. After 7 days, one aliquot of each strain was thawed, and a colony forming unit (CFU) assay was used to determine the bacteria number.

On the day of the experiment, *M. marinum* strains were thawed, washed in RPMI 1640 medium, and then suspended in culture medium without P/S. Then, clumps of bacteria were disrupted by multiple passages through a 25-gauge needle, and serial dilutions of bacteria in culture medium without P/S were prepared.

### THP-1 cell culture

The human monocyte-macrophage cell line, THP-1 cells (ACTC TIB-202; American Type Culture Collection, Manassas, VA, USA) were used. THP-1 cells were maintained in culture medium composed of RPMI 1640 medium supplemented with 1 mM sodium pyruvate, 10% FBS, 0.05 mM β-mercaptoethanol and antibiotics—100 U/ml of penicillin and 100 μg/ml of streptomycin. The cells were passaged every 3 days.

### Phagocytosis and intracellular growth of bacteria

THP-1 cells (monocytes) suspended in culture medium (see above) were distributed to 24-well plates (Nunc, Roskilde, Denmark) at the density of 10^5^ cells/well and differentiated into macrophages during 24 h of culture with 20 ng/ml of PMA (37 °C; 5% CO_2_). The ability of macrophages to attach to the plastic surface of the plates was confirmed by light microscopy [48]. After replacement of culture medium without antibiotics macrophages were pretreated with 35 μg/ml of anti-TLR2 blocking monoclonal antibodies (mAb) or with 55 μg/ml of anti-CR3 blocking mAb or appropriate isotype control for 1 h (37 °C, 5% CO_2_) or left untreated. The mAb at the utilized concentrations were sufficient to blocking surface expression of each receptor as was described previously [49]. Prior to infection, the macrophages were equilibrated at 33 °C, 5% CO_2_ for 1 h according to Subbian et al. [50]. Next, phagocytes were infected with wild-type *M. marinum*, its mutants (*MMAR_2343*::*Tn*, Δ*MMAR_2349*, Δ*MMAR_2321* or Δ*MMAR_2331*) or complemented strains (Δ*MMAR_2349*_C, Δ*MMAR_2321_C* or Δ*MMAR_2331_C*) at a 1:10 multiplicity of infection (MOI) and incubated for 2 h (33 °C, 5% CO_2_). Next, in order to remove non-ingested bacilli, macrophages were extensively washed three times with HBSS and then, to kill extracellular bacteria, phagocytes were incubated in culture medium containing 1 mg/ml of gentamycin for 1 h (37 °C, 5% CO_2_). The gentamycin concentration used in the experiment was determined in preliminary study and confirmed to be bactericidal towards *M. marinum*. After incubation, macrophages were washed two times with HBSS, and then fresh culture medium without antibiotics as well as anti-TLR2 blocking mAb or anti-CR3 blocking mAb (where required) were added, and infected phagocytes were cultured for 2 days.

On the day of infection (day 0) and 2 days post-infection, the macrophages were lysed with 1 ml of 0.2% Triton X-100 for 30 min, on ice. Appropriate dilutions of cell lysates were prepared and plated onto Middlebrook 7H10 agar supplemented with 10% of OADC. After 14 days of incubation (30 °C), the colony number was counted. For phagocytosis, the data are presented as CFUs/ml from day 0. For intracellular growth of bacteria, the data are presented as fold-increase in CFUs/ml, calculated as (CFUs/ml on day 2)/(CFUs/ml on day 0).

### TLR2 and CR3 expression on macrophages infected with *M. marinum* strains

THP-1 cells in culture medium were distributed into 6-well plate (Nunc) at the density of 3 × 10^6^ cells/well and differentiated into macrophages with PMA as described above and then fresh culture medium without antibiotics was added. Prior to infection, macrophages were equilibrated at 33 °C, 5% CO_2_ for 1 h. Next, phagocytes were infected with wild-type *M. marinum* or its mutants, *MMAR_2343*::*Tn*, Δ*MMAR_2349*, Δ*MMAR_2321* and Δ*MMAR_2331*, at a 1:10 MOI for 2 h (33 °C, 5% CO_2_). Then, the non-ingested bacteria were removed by extensive washing of the macrophages with HBSS. Subsequently, phagocytes with engulfed bacteria were harvested, centrifuged (130*g*, 8 min) and incubated in 4% FA for 30 min at 4 °C. Thereafter, the macrophages were washed once in D-PBS supplemented with 1% FBS. Before staining with the mAbs, crystallizable fragment receptors (FcRs) were blocked using D-PBS with 10% human AB serum for 15 min at room temperature to prevent non-specific antibody binding. Afterwards, the cells were washed twice in D-PBS with 1% FBS and stained with 5 μl PE-conjugated anti-TLR2 mAb, 20 μl PE-conjugated anti-CR3 mAb or 10 μl of the appropriate isotype control for 30 min at 4 °C. Then, the cells were washed twice in D-PBS with 1% FBS, suspended in D-PBS and analyzed at once with an FACS LSR II BD flow cytometer (Becton–Dickinson, USA) that was equipped with the BD FACS Diva Software. The results are presented as median fluorescence intensity (MFI), which correlates with the surface expression of the target molecule.

### Production of ROS and NO by macrophages treated with blocking antibodies

ROS production was measured using a luminol-enhanced chemiluminescence method. THP-1 cells in culture medium were distributed into 96-well black plate (Nunc) at the density of 1 × 10^5^ cells/well and differentiated into macrophages (see above). Then, culture medium was removed and macrophages were pretreated with anti-TLR2 or anti-CR3 blocking monoclonal antibodies in HBSS as described above or cells left untreated. Thereafter, 1 μg/ml PMA (to initiate the oxygen burst) and 1 mM luminol and 40 U horseradish peroxidase (to enhance chemiluminescence were added to macrophages. Chemiluminescence was measured during 4 h at 5-min intervals using a Fluoroscan Ascent FL instrument (Labsystems, Helsinki, Finland). Data were acquired as relative light units (RLU), and the area under the curve of chemiluminescence versus assay time (total RLU) was calculated.

Production of NO by macrophages was determined using the Griess reagent (1:1 mix of 0.1% *N*-(1-naphthyl)ethylenediamine dihydrochloride and 1% sulfanilic acid in 5% phosphoric acid) which detect a nitrite, a stable metabolite of NO. Briefly, obtained macrophages (1 × 10^5^ cells/well in a 96-well plate, NUNC) were treated with blocking monoclonal antibodies in culture medium for 48 h (36 °C, 5% CO_2_). The absorbance of culture supernatants was measured on a Multiscan RC ELISA reader (Labsystems) at wavelength of 550 nm. Nitrite concentration was calculated from a standard curve using sodium nitrite as a reference. The data were presented as nitrite concentration (μM).

## Statistical analysis

The statistical evaluation of the results, which is presented as the mean ± SEM, was performed using data from 5 to 6 independent experiments. Statistical significance was verified using the Wilcoxon’s singed rank and Mann–Whitney *U* tests. Statistica 10.0 software was used for statistical calculations, and the statistical significance was defined as p ≤ 0.05.
